# An Environmentally Tolerant 5A Hydrogel with Photothermal Effect for Frostbite Treatment

**DOI:** 10.3390/gels12060554

**Published:** 2026-06-20

**Authors:** Jianmei Chen, Yifan Wu, Tiantian Zhu, Hongyu Wu, Meiling Su, Zongguang Liu

**Affiliations:** 1 Key Laboratory of the Jiangsu Higher Education Institutions for Integrated Traditional Chinese and Western Medicine in Senile Diseases Control, School of Traditional Chinese Medicine, Faculty of Medicine, Yangzhou University, Yangzhou 225009, China; cjm@yzu.edu.cn (J.C.);; 2 Microelectronics Industry Research Institute, School of Physics Science and Technology (Integrated Circuit Science and Engineering), Yangzhou University, Yangzhou 225009, China

**Keywords:** hydrogel, environmentally tolerant, photothermal effect, anti-freezing, frostbite

## Abstract

Rapid rewarming is the most conventional and primary treatment for frostbite, yet effective adjunctive strategies remain absent. Conventional wound dressings, such as therapeutic hydrogels, tend to freeze and lack the necessary rewarming ability, rendering them unsuitable for direct application. Herein, we engineered an environmentally tolerant photothermal hydrogel, named 5A-Gel, featuring anti-swelling, anti-pressure, antioxidant, anti-freezing, and anti-drying capacities, for the treatment of frostbite. 5A-Gel was formed via dynamic crosslinking between gelatin and tea polyphenols in a glycerol/water solvent system. The incorporation of glycerol endowed the hydrogel with superior anti-swelling, anti-freezing, and anti-drying performance (remaining flexible at −20 °C and 37 °C for at least 60 days), along with concentration-dependent antioxidant activity due to tea polyphenols. Furthermore, 5A-Gel exhibited excellent photothermal effects, maintaining stable temperature and softness under 808 nm laser irradiation with robust cyclic durability. In addition, 5A-Gel showed slow degradability, excellent hemocompatibility, and favorable in vivo biosafety. Functionally, in a mouse frostbite wound model, photothermal rewarming therapy using 5A-Gel markedly expedited frostbite healing, promoting re-epithelialization, enhancing collagen deposition, alleviating inflammatory response, and stimulating neovascularization. Therefore, the as-prepared 5A-Gel serves as a competent therapeutic platform for in situ frostbite treatment and offers innovative principles for the rational engineering of high-performance hydrogel systems targeting frostbite tissue injuries.

## 1. Introduction

Frostbite constitutes a major cold-induced injury, especially among those involved in high-altitude activities, polar expeditions, and certain military operations [1,2]. Pathophysiologically, it results from prolonged exposure to extremely low temperatures, encompassing ice crystal formation within tissues, microvascular occlusion, and an inflammatory cascade that ultimately leads to tissue necrosis in the absence of timely and adequate intervention [2]. Notably, severe frostbite can result in irreversible tissue necrosis and even amputation, presenting a formidable clinical hurdle [1,3]. Therefore, effective therapeutic strategies for frostbite are imperative. Currently, the established therapeutic approach for frostbite is rapid rewarming, typically by immersing the affected area in warm water (37–42 °C), which facilitates restoration of blood circulation and limits tissue damage [4,5]. Nevertheless, rapid rewarming is frequently hindered in remote settings, challenging to maintain precise thermal regulation, and provides no durable post-rewarming defense, thereby exacerbating secondary injury, oxidative stress, and impaired healing [4,6,7]. Hence, developing adjuvant treatment strategies is urgently required to simultaneously realize efficient rewarming, durable tissue protection and lasting curative functions.

Photothermal therapy has recently emerged as a promising alternative to water rewarming, offering localized, on-demand heating with higher efficacy and safety, and holding great potential in wound healing as well as frostbite treatment [6,8,9,10]. When integrated into hydrogels, which possess high water content, good hydrophilicity, biocompatibility, and porous structures similar to the extracellular matrix, photothermal therapy enables localized, on-demand heating for wound management [11,12]. Multiple photothermal agents, including polydopamine, MXene, and gold nanoparticles, have been integrated into hydrogel matrices to implement therapeutic interventions against infectious or diabetic cutaneous wounds. Nevertheless, most of these hydrogels are designed for use at room temperature and are ill-suited for frostbite therapy in cold environments. These hydrogels display weak environmental resistance originating from excessive internal water and hydrophilic crosslinked frameworks, rendering them prone to adverse swelling, ice crystal accumulation under freezing environments, and structural shrinkage upon water loss [13,14,15,16]. Furthermore, frostbite wounds are often accompanied by excessive reactive oxygen species (ROS), but few existing hydrogels simultaneously address all the requirements for frostbite [10,17].

To overcome these drawbacks, a multifunctional photothermal hydrogel with robust environmental adaptability was rationally engineered, which unifies outstanding photothermal conversion performance and five favorable functional features, including anti-swelling, anti-compression, anti-freezing, anti-drying and antioxidant capabilities, thereby designated as 5A-Gel. Specifically, the hydrogel was fabricated by dynamic crosslinking between gelatin and tea polyphenols (TPP) within a glycerol/water binary solvent system, with sodium periodate (NaIO_4_) acting as the reaction initiator [18]. Glycerol serves as both a cryoprotectant and a moisturizer, forming abundant hydrogen bonds with water to prevent ice crystal formation and water evaporation [19,20]. Cumulative research progress has validated the potential of glycerol-containing antifreeze hydrogels for wound management under harsh ambient conditions [21]. Tea polyphenols, natural antioxidants rich in phenolic hydroxyl groups, endow the hydrogel with ROS scavenging ability and have been widely incorporated into hydrogel dressings for antioxidant, anti-inflammatory, and wound healing applications [22]. The resulting 5A-Gel is expected to remain flexible at subzero temperatures, resist dehydration under warm conditions, efficiently scavenge free radicals, and generate controlled heat upon Near Infrared (NIR) irradiation. Subsequent in vitro and in vivo evaluations confirm its degradability, hemocompatibility, and biosafety. Most importantly, in a mouse frostbite wound model, 5A-Gel combined with photothermal rewarming significantly accelerates wound healing by promoting re-epithelialization and collagen remodeling, reducing inflammation, and enhancing neovascularization. Taken together, the present study offers a feasible and efficient approach for on-site frostbite therapy under low-temperature conditions and paves a new route to fabricate multi-functional hydrogels targeting cold-triggered tissue damage.

## 2. Results and Discussion

### 2.1. Fabrication of 5A-Gel

**Figure 1:**
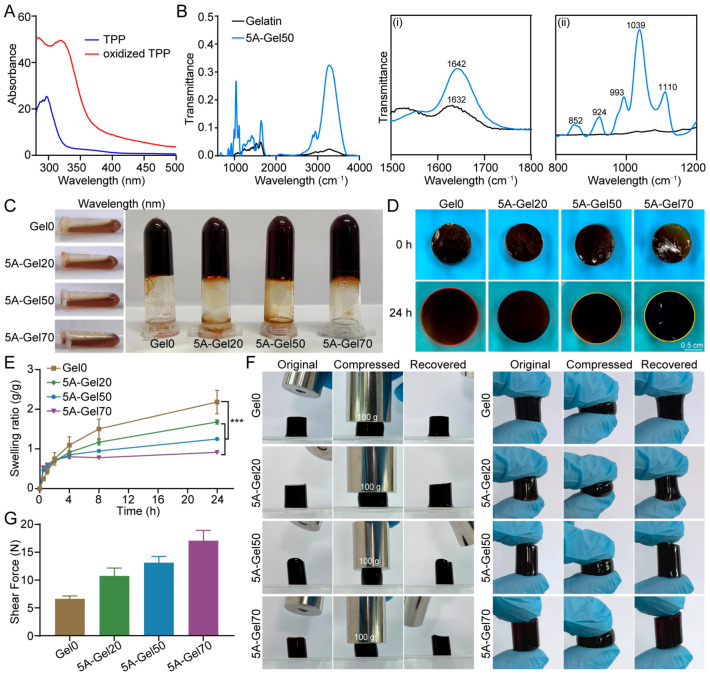
Preparation and characterization of Gel0 and 5A-Gel: (**A**) UV–Vis spectra of TPP and oxidized TPP. (**B**) FTIR spectra of gelatin and 5A-Gel50. The enlarged images in the ranges of (**i**) 1500–1800 and (**ii**) 800–1200 cm^−1^. (**C**) Macroscopic photographs of gelation of Gel0 and 5A-Gel. (**D**) Morphological changes in Gel0 and 5A-Gel before and after 24 h incubation in PBS at 37 °C. (**E**) Weight swelling ratios of Gel0 and 5A-Gel in PBS at 37 °C. *** *p* < 0.001. (**F**) Compression performance of Gel0 and 5A-Gel under a 100 g weight and squeezing with fingers. (**G**) Shear resistance of Gel0 and 5A-Gel.

To synthesize the 5A-Gel series, gelatin and TPP were blended in glycerol/water solutions with glycerol gradients of 20, 50, and 70% *v*/*v*. Subsequently, NaIO_4_ was introduced to oxidize TPP into TPP quinone. Mechanistically, in neutral or alkaline environments, TPP undergoes conversion into its oxidized form, producing quinone moieties, which can covalently interact with nucleophilic amino residues of gelatin via Schiff base or Michael addition reactions, forming robust crosslinking networks [18,23]. The oxidation of TPP to TPP quinone was verified by UV–Vis spectroscopy, presenting an obvious absorbance rise near 340 nm (Figure 1A), consistent with polyphenol oxidation to quinones [24,25].

To clarify the internal crosslinking pathway of the as-prepared hydrogel, FTIR measurements were conducted. Figure 1B illustrates that, relative to pristine gelatin, the 5A-Gel50 spectrum showed typical absorption features at 852, 924, 993, 1039 and 1110 cm^−1^, corresponding to the characteristic peaks of glycerol [26]. In 5A-Gel50, the band near 3300 cm^−1^ appeared to be wider than that in gelatin, indicating strong intermolecular hydrogen bonds between gelatin chains and polyphenolic compounds [27]. Notably, the peak attributed to the C=N stretching vibration (1632 cm^−1^) broadened and shifted to a higher wavenumber (1642 cm^−1^) in the 5A-Gel50 spectra, confirming the occurrence of Schiff base reactions involving gelatin and TPP [28]. Simultaneously, the amide III band moved from 1236 cm^−1^ in gelatin to 1238 cm^−1^ in 5A-Gel50, and the peak at 1451 cm^−1^ (originating from aromatic C-N stretching or CH_2_ bending modes) became more intense and moved to higher frequencies (1455 cm^−1^) in 5A-Gel50, caused by the Michael addition reaction between TPP and gelatin [28,29]. Therefore, the hydrogel network is jointly stabilized by non-covalent hydrogen bonds and covalent crosslinks including Schiff base and Michael addition bonds.

The resultant hydrogels were named as 5A-Gel20, 5A-Gel50, and 5A-Gel70 according to their respective glycerol concentrations (20%, 50%, and 70% *v*/*v*). For comparison, a control hydrogel (Gel0) was prepared using the same protocol, except that pure water was used as the solvent for gelatin and TPP crosslinking instead of the glycerol/water mixture. Figure 1C presents the macroscopic appearance of these hydrogels along with their corresponding designations.

### 2.2. Anti-Swelling Property of 5A-Gel

Due to abundant hydrophilic groups, most hydrogels undergo obvious dimensional swelling when immersed in aqueous media. Uncontrolled volumetric swelling exerts adverse impacts on the materials’ mechanical robustness and other functional characteristics, which drastically narrows the range of available usage scenarios [30]. The swelling behavior of the 5A-Gel series was tested in phosphate-buffered saline (PBS). As depicted in Figure 1D, all tested hydrogels retained their shape after 24 h of immersion. Notably, after incubation, Gel0 exhibited a visibly swollen morphology compared to 5A-Gel20, 5A-Gel50, and 5A-Gel70. Quantitatively, after 24 h, 5A-Gel20, 5A-Gel50 and 5A-Gel70 increased by 40%, 28% and 26% in their original diameters, respectively. This enhanced anti-swelling property originates from extensive intermolecular hydrogen bonding generated between glycerol and water molecules, a mechanism that restrains excessive liquid absorption [19,20].

Weight change measurements provided a quantitative assessment of the swelling ratios (Figure 1E). A rapid swelling phase occurred within the initial 4 h, a feature desirable for applications requiring immediate fluid uptake. Subsequent incubation revealed clear differences among the groups. Specifically, 5A-Gel20, 5A-Gel50, and 5A-Gel70 attained swelling ratios of 168%, 125%, and 91%, respectively. Comparatively, Gel0 showed a progressively increasing swelling ratio (218%). Collectively, these data demonstrate that all 5A-Gel formulations exhibit remarkable resistance to excessive water uptake, rendering these materials highly appropriate for scenarios requiring regulated water absorption and steady geometric integrity, including tissue regeneration and cutaneous wound repair.

### 2.3. Anti-Compression Performance of 5A-Gel

As shown in Figure 1F, all hydrogel groups exhibited compressibility and resilience without fracture under a 100 g weight or finger pressure, and they rapidly recovered to their initial state after unloading, demonstrating notable compression resistance behavior and excellent self-recovery performance. Furthermore, the mechanical properties improved with increasing glycerol concentration, as evidenced by the shear forces: compared with Gel0, 5A-Gel20, 5A-Gel50, and 5A-Gel70 showed progressively higher shear forces (Figure 1G). The improved mechanical properties can be attributed to an interpenetrating dual-network structure, where glycerol incorporation enables noncovalent networks to integrate with the gelatin-TPP crosslinked networks [21]. When the hydrogel was pressed, the long-chain polymer network remained intact and stabilized the deformation.

### 2.4. Anti-Freezing and Anti-Drying Properties of 5A-Gel

Conventional hydrogels tend to freeze readily at low temperatures, which poses a severe limitation to their practical applications in cold environments [13,14,15,16]. Glycerol, a prevalent biocompatible humectant and cryoprotective additive, can generate robust hydrogen-bonded networks with water [19,20]. This interaction not only suppresses water evaporation during thermal exposure but, more importantly, disrupts the formation of ice crystal lattices at subzero temperatures, thereby endowing hydrogels with freezing tolerance.

**Figure 2:**
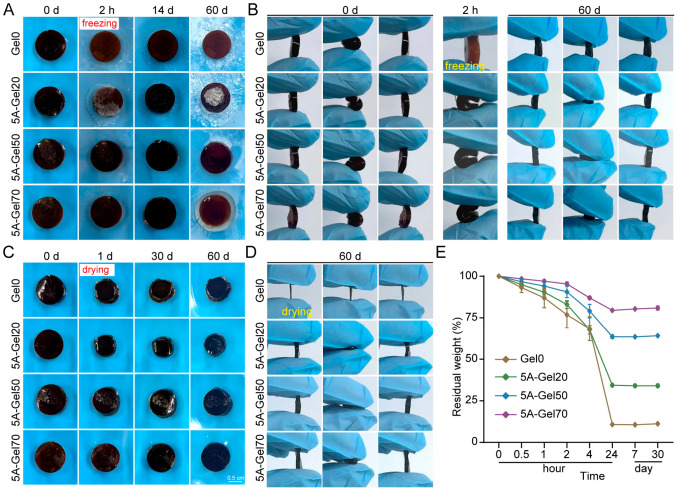
Anti-freezing and anti-drying properties of 5A-Gel: (**A**) Macroscopic images of Gel0 and 5A-Gel stored at −20 °C for predefined time periods. (**B**) The flexibility of Gel0 and 5A-Gel stored at −20 °C at 0 h, 2 h and 60 d. (**C**) Macroscopic images of Gel0 and 5A-Gel stored at 37 °C for predefined time periods. (**D**) The flexibility of Gel0 and 5A-Gel stored at 37 °C after 60 d. (**E**) Weight changes in Gel0 and 5A-Gel stored at 37 °C.

To evaluate the anti-freezing performance, hydrogels with varying glycerol contents (Gel0, 5A-Gel20, 5A-Gel50, and 5A-Gel70) were stored at −20 °C. As shown in Figure 2A, Gel0 froze solid within 2 h, while 5A-Gel20 only exhibited surface frost. By contrast, both 5A-Gel50 and 5A-Gel70 remained intact for at least 60 days. Notably, glycerol leaching appeared on the surface of 5A-Gel70 starting from day 14, indicating phase separation due to excess glycerol content [31].

Mechanical tests were performed immediately after removing the samples from the freezer at predetermined time points. Throughout the entire storage period, 5A-Gel20, 5A-Gel50, and 5A-Gel70 remained flexible and could be extensively twisted without damage, quickly bouncing back to their original shape (Figure 2B). This manual bending test directly demonstrates the anti-freezing behavior: the glycerol-induced freezing point depression prevents ice crystallization, thereby preserving the hydrogel’s flexibility at −20 °C. Taken together, these results confirm that glycerol acts as a simple yet effective additive to ensure reliable hydrogel operation in frozen conditions.

To evaluate water retention, hydrogels with varying glycerol concentrations were incubated at 37 °C. As shown in Figure 2C, Gel0 turned into a hard and shrunken polymer, while all glycerol-containing hydrogels (5A-Gel20, 5A-Gel50 and 5A-Gel70) remained moist and structurally intact for at least 60 days. Figure 2D,E further reveal that Gel0 lost weight quickly and became stiff within 24 h, whereas 5A-Gel20, 5A-Gel50, and 5A-Gel70 retained high flexibility and excellent weight retention throughout the entire period. These results verify that the incorporation of glycerol can markedly boost the hydrogels’ capacity to resist dehydration.

### 2.5. Antioxidant Properties of 5A-Gel

**Figure 3:**
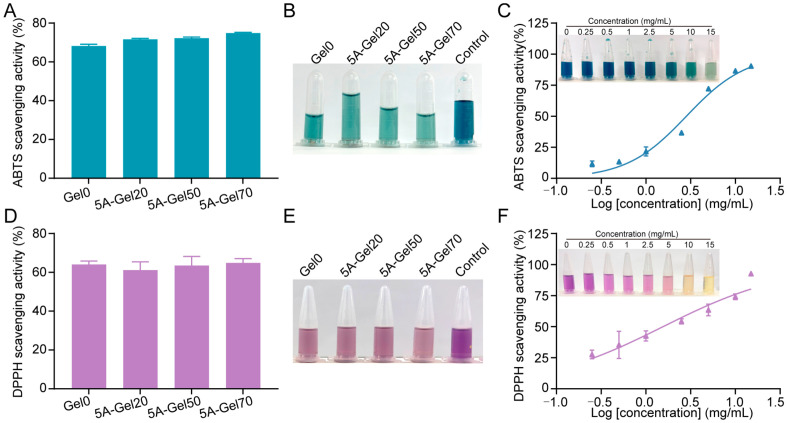
Antioxidant capacity of Gel0 and 5A-Gel: (**A**) Quantitative scavenging ability of Gel0 and 5A-Gel against ABTS radicals. (**B**) Visible color change in ABTS+ solution following a 30 min incubation period. (**C**) Quantitative scavenging ability of 5A-Gel50 against ABTS radicals. Inset: Color change in ABTS+·solution. (**D**) Scavenging ability of Gel0 and 5A-Gel against DPPH. (**E**) Color change in DPPH solution. (**F**) Scavenging ability of 5A-Gel50 against DPPH. Inset: Color change in DPPH solution.

Wound cover hydrogels possessing powerful ROS neutralizing capacity are capable of facilitating skin wound repair. The free radical elimination capacity of 5A-Gel was tested using 2,2′-azino-bis(3-ethylbenzothiazoline-6-sulfonic acid) (ABTS) and 2,2-diphenyl-1-picrylhydrazyl (DPPH) radical scavenging tests, providing preliminary evidence of antioxidant potential. As shown in Figure 3A, all the hydrogels exhibited outstanding clearance performance toward ABTS free radicals, which could also be visualized through the discoloration of ABTS working fluid after hydrogel specimens were immersed (Figure 3B). The correlation between 5A-Gel50 concentration and radical scavenging ratio further validated the dose-responsive antioxidative behavior (Figure 3C). Consistently, the blue-green ABTS+·solution turned progressively transparent once 5A-Gel50 was introduced into the system (Figure 3C, inset).

DPPH radical scavenging followed a comparable pattern. As presented in Figure 3D, all tested hydrogels efficiently quenched DPPH radicals, with the solution color shifting from deep purple to pale yellow after hydrogel addition (Figure 3E). The concentration-dependent activity of 5A-Gel50 was further confirmed (Figure 3F), and increasing amounts of the hydrogel progressively decolorized the purple DPPH solution (Figure 3F, inset). Based on the covalent crosslinking chemistry (Schiff base and Michael addition) that immobilizes most TPP derivatives within the gelatin network, we propose that the antioxidant activity primarily originates from the remaining free phenolic hydroxyl groups on the immobilized structures, a notion supported by the literature [32,33].

### 2.6. Photothermal Properties

**Figure 4:**
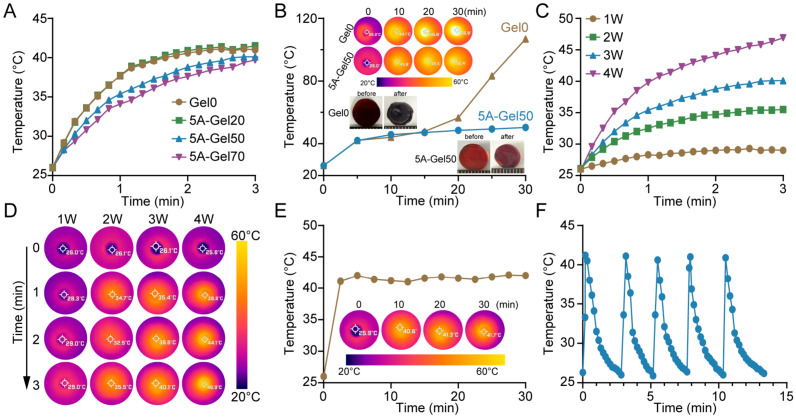
Photothermal properties of Gel0 and 5A-Gel: (**A**) The real-time temperature variation curves of Gel0 and 5A-Gel under 808 nm irradiation. (**B**) The temperature variation curves of Gel0 and 5A-Gel50 under 808 nm irradiation (3 W) for 30 min. (**C**) The temperature variation curves and (**D**) corresponding infrared thermal images of 5A-Gel50 under 808 nm irradiation at various power densities for 3 min. (**E**) The temperature variation curve and corresponding infrared thermal image of 5A-Gel50 with fine-tuned laser power to maintain ~41 °C for 30 min. (**F**) The heating and cooling curves for five laser on–off cycles of 5A-Gel50.

The conjugated quinone structures and intermolecular π–π stacking of TPP quinone generate a broad absorption tail into the NIR region, accounting for its photothermal effect under 808 nm irradiation [25,34]. The photothermal conversion capability of the hydrogels was evaluated. As shown in Figure 4A, under 808 nm irradiation (3 W), all the hydrogels exhibited a rapid temperature increase, reaching ~40 °C within 3 min. The temperature changes in Gel0 and 5A-Gel50 were monitored during 30 min of 808 nm irradiation at 3 W. As shown in Figure 4B, Gel0 displayed a drastic temperature elevation, rising from room temperature to 106 °C. This dramatic temperature rise is due to the absence of glycerol in Gel0. Under prolonged NIR irradiation, Gel0 rapidly loses water and becomes completely dry (Figure 4B, inset), leading to a drastic reduction in heat capacity and loss of evaporative cooling, which causes continuous heat accumulation. In contrast, although the temperature of 5A-Gel50 gradually increased over time due to a slow thermal accumulation effect, it reached only ~50 °C and remained soft after irradiation (Figure 4B, inset). 5A-Gel50 contains 50% glycerol, which retains water through hydrogen bonding, thereby limiting the equilibrium temperature.

The temperature response of 5A-Gel50 is power-dependent, with higher power densities triggering faster and higher temperature rises (Figure 4C,D). Leveraging this power-dependent behavior for practical applications, the temperature of 5A-Gel50 could be maintained precisely at ~41 °C for at least 30 min by fine-tuning the laser power, demonstrating excellent controllability and promising application prospects in photothermal therapy (Figure 4E). Moreover, 5A-Gel50 exhibited remarkable photothermal cycling stability, with consistent heating and cooling curves over five consecutive on–off cycles (Figure 4F), confirming its reusability and structural robustness under repeated irradiation. This stable photothermal performance under repeated irradiation is desirable for potential clinical applications requiring multiple treatment sessions. While the present work demonstrates the excellent, controllable and stable photothermal effect, quantitative evaluation of photothermal conversion efficiency will be pursued in future studies.

### 2.7. Degradability and Biosafety of Gel0 and 5A-Gel

**Figure 5:**
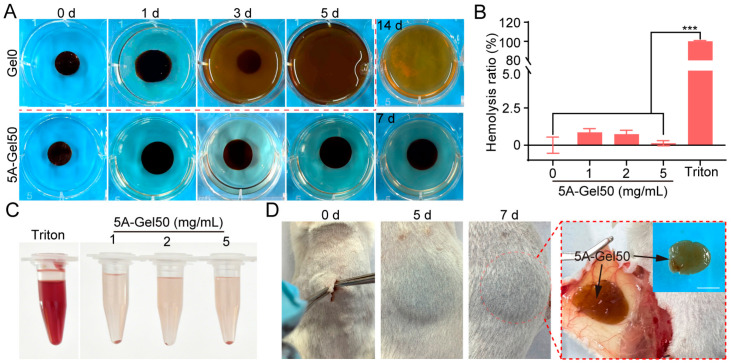
Degradability and biosafety of Gel0 and 5A-Gel: (**A**) Stability of 5A-Gel50 in PBS at 37 °C over 14 days. (**B**) Hemolysis ratios of 5A-Gel50. *** *p* < 0.001. (**C**) Photographs of centrifuge tubes after hemolysis test. (**D**) Subcutaneous implantation of 5A-Gel in mice after 7 days. (Inset shows the hydrogel peeled from the mouse).

Given that biodegradability plays a vital role in biomedical applications, the stability of 5A-Gel was investigated in physiological ambient surroundings (PBS, pH 7.4, 37 °C). As shown in Figure 5A, the hydrogel exhibited slow degradability over 14 days, which is desirable for sustained wound protection and tissue regeneration.

Given that frostbite is often accompanied by hemorrhage, the hemocompatibility of 5A-Gel50 was analyzed. As illustrated in Figure 5B,C, the hemolysis ratios of the 5A-Gel50-treated groups were calculated to be <1% based on the absorbance at 570 nm, indicating that the hydrogel meets the requirement for non-hemolytic biomaterials. After centrifugation, the Triton-treated positive group showed a homogeneous red liquid in the tube, indicating complete red blood cell lysis. In contrast, in the experimental groups, the supernatants were almost colorless, and red blood cells were deposited at the bottom of the tubes, visually confirming the hemocompatibility of 5A-Gel50.

The in vivo biosafety of 5A-Gel50 was further assessed by subcutaneous implantation. As presented in Figure 5D, during the 7-day implantation period, the surrounding skin remained normal without any signs of irritation or inflammation, and the hydrogel maintained excellent stability in the subcutaneous environment. Taken together, these results support rudimentary acute safety outcomes of 5A-Gel50 and demonstrate its potential for further therapeutic evaluation in frostbite treatment. Future studies should focus on systematic evaluation of cytocompatibility and peri-implant tissue histology staining to further validate the biosafety of 5A-Gel50 before clinical translation.

### 2.8. Therapeutic Effects of 5A-Gel50 on Frostbite Wound

**Figure 6:**
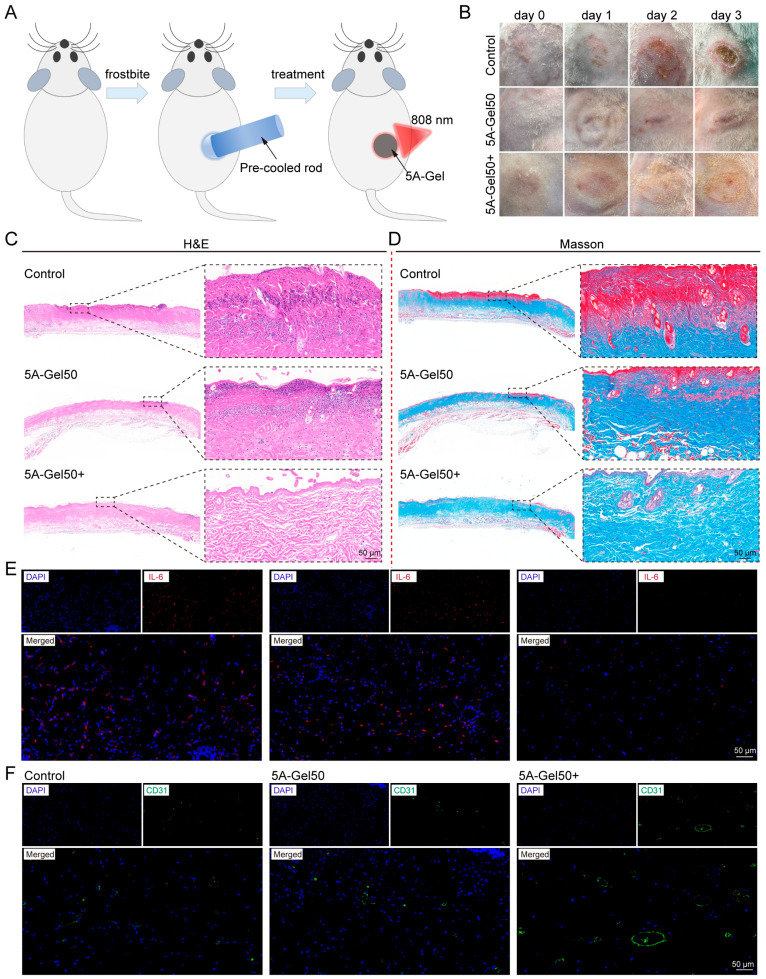
Frostbite wound healing effects of 5A-Gel50: (**A**) Schematic procedure of establishing mouse frostbite injury models. (**B**) The photographs of the wounds on Days 0, 1, 2, and 3. (**C**) Representative H&E staining of the frostbite model mice at day 3. The dashed box indicates a local enlargement. (**D**) Masson’s trichrome staining. (**E**) Immunofluorescent staining of IL-6. (**F**) Immunofluorescent staining of CD31.

It is well accepted that rapid rewarming at 37–42 °C for 15–30 min promotes blood circulation and tissue repair, making it the most common approach for frostbite treatment [4,5]. Motivated by the preceding findings, we established a mouse frostbite model to test the healing effect of 5A-Gel50, either alone or combined with photothermal rewarming using an 808 nm laser (Figure 6A). For the photothermal-treated group (5A-Gel50+), the hydrogel was covered on the frostbite wound and irradiated with an 808 nm laser to maintain 41 ± 1 °C for 30 min. Wound status in each group was documented photographically during treatment.

At day 3 after injury, the frostbite wound was smallest and least severe in the 5A-Gel50+ group compared to the control and 5A-Gel alone groups (Figure 6B), suggesting that photothermal therapy contributed remarkably to accelerating healing. The 5A-Gel50 alone group showed moderate improvement, likely due to the hydrogel’s moisturizing and antioxidant effects [8], while the significantly better outcomes in the 5A-Gel50+ group indicate the dominant role of photothermal rewarming. Overall, 5A-Gel50 exhibited excellent healing effects on frostbite wounds.

Moreover, the histopathology of frostbite healing was analyzed using H&E, Masson’s trichrome, and immunofluorescence staining. The H&E staining images of both the control group and the 5A-Gel50 group showed an unrepaired epidermis, thickened granulation tissue, disordered arrangement of collagen fibers, and mild inflammatory cell infiltration. In contrast, in the 5A-Gel50+ group, the epidermis was notably intact with a continuous stratum corneum. The collagen fibers were arranged in a regular, parallel orientation based on blinded morphological observation, indicating effective tissue remodeling. Importantly, only a few scattered inflammatory cells were observed in the dermis, suggesting a significant reduction in inflammation. These histological findings indicate that the combination of 5A-Gel50 with photothermal rewarming effectively promotes epidermal regeneration, reduces inflammatory responses, and accelerates the reconstruction of dermal tissue.

Furthermore, Masson’s trichrome staining was carried out to assess collagen accumulation and tissue structural organization (Figure 6D). In the control group, severe structural disintegration was observed, accompanied by extensive red-stained areas that indicated massive inflammatory infiltration. The 5A-Gel50-treated group showed marked improvement, characterized by reduced inflammation and increased collagen deposition; however, the fibers appeared dense and compact, suggesting robust but immature matrix remodeling. In contrast, the 5A-Gel50+ group achieved superior regeneration, displaying a highly organized architecture with regularly arranged, wavy collagen bundles, alongside minimal residual inflammation and a continuous epidermal layer, confirming a high-quality healing process.

To further investigate inflammation and angiogenesis, immunofluorescence staining for IL-6 and CD31 was conducted. As shown in Figure 6E, the fluorescence intensity of IL-6 was markedly diminished in the 5A-Gel50+ group relative to both the control and 5A-Gel50 groups, supporting the H&E findings of reduced inflammation. For vascularization, the 5A-Gel50+ group exhibited a higher vessel density (103 ± 16/mm^2^) than that of 5A-Gel (70 ± 25/mm^2^) and control (68 ± 15/mm^2^) groups based on the endothelial cell marker CD31 staining (Figure 6F), suggesting a significant enhancement in neovascularization. Collectively, these results demonstrate that the combination of 5A-Gel50 with photothermal therapy not only accelerates the resolution of inflammation but also robustly promotes vascular regeneration, thereby synergistically facilitating frostbite wound healing. The wound-healing study was terminated at day 3 post-injury, a limitation that does not allow assessment of long-term tissue remodeling, scar formation, or complete wound closure. Longer observation periods will be included in future studies.

## 3. Conclusions

In summary, we successfully developed an environmentally tolerant 5A-Gel via dynamic crosslinking of gelatin and TPP in a glycerol/water system. The incorporation of glycerol conferred excellent anti-freezing and anti-drying capability. The hydrogels also demonstrated concentration-dependent antioxidant activity, along with robust photothermal conversion that enabled precise temperature control. In addition, 5A-Gel displayed slow degradability, good hemocompatibility, and favorable biosafety. Notably, in a mouse frostbite model, 5A-Gel combined with photothermal rewarming substantially expedited wound healing, as reflected by improved re-epithelialization, well-organized collagen deposition, reduced inflammation, and enhanced neovascularization. Taken together, 5A-Gel provides an effective all-in-one platform for frostbite treatment in cold environments, integrating anti-freezing, antioxidant, photothermal, and biocompatible functions.

## 4. Materials and Methods

Materials: Gelatin, TPP and NaIO_4_ were sourced from Aladdin (Shanghai, China). ABTS was bought from Kaiwei Chemical (Shanghai, China). DPPH was acquired from Macklin (Shanghai, China). PBS was obtained from KeyGen (Nanjing, Jiangsu, China).

Hydrogel Preparation: TPP powder was homogeneously dispersed at a loading level of 20 wt% (by mass relative to gelatin) into a 10% (*w*/*v*) gelatin aqueous solution containing different glycerol-to-water volumetric proportions (0/100, 20/80, 50/50, or 70/30 *v*/*v*). Oxidative covalent crosslinking was triggered by the addition of NaIO_4_. The resultant mixture was subsequently shaken at high rotation speed for 30 s to yield a homogeneous mixture, followed by incubation inside a water bath pre-set to 37 °C to facilitate complete gel network formation.

FTIR and UV–Vis spectroscopy: The molecular framework of the prepared hydrogel materials was analyzed via Fourier transform infrared (FTIR) spectroscopy using a Bruker ALPHA II instrument (Bruker, Germany), with spectral scans collected across the wavenumber range of 400–4000 cm^−1^. The optical absorption spectra of TPP before and after oxidation treatment with NaIO_4_ were collected within the wavelength range of 200–500 nm.

Swelling Test: Pre-weighed hydrogel samples (W_0_) were soaked in PBS maintained at 37 °C for set periods. At predefined time intervals, the samples were extracted and subjected to mass recording (W_t_). The swelling ratio (SR) was calculated using the formula below:

SR (%) = (W_t_ − W_0_) × 100/W_0_ (1)

Anti-drying Property Assessment: To evaluate drying resistance, water retention capacity was tracked. Samples were initially weighed (W_0_) before being placed in a thermostatic incubator maintained at 37 °C for a maximum period of 60 days. At various time points, the samples were taken out and promptly weighed using a high-precision analytical balance. The residual weight percentage (RW) was determined via the calculation below:

RW (%) = W_t_ × 100/W_0_ (2)

in which W_t_ corresponds to the sample mass collected at time point t.

Anti-freezing Property Assessment: Low-temperature tolerance was characterized by placing cylindrical samples (~8 mm in diameter) inside an uncovered 6-well cell culture plate and leaving them at −20 °C. At predetermined time points (spanning 0 h up to 60 days), all specimens were taken out and instantly folded by hand to assess their flexibility and integrity.

Antioxidant Activity Evaluation: ABTS (7 mM) with potassium persulfate (2.5 mM) was mixed in ultrapure water to generate the ABTS working solution, followed by a 16 h reaction in the dark at room temperature. Before the test, the solution was adjusted to an absorbance of 0.70 ± 0.02 at 734 nm with ethanol. For assessing antioxidant activity, each pre-weighed hydrogel sample was submerged into the ABTS working solution and maintained at 37 °C in darkness. As a blank control, a sample containing only the working solution was run simultaneously. After a 30 min incubation, the reaction mixture (100 μL) was withdrawn from each sample, and its absorbance at 734 nm was recorded using a microplate reader (Bio-Tek SYNERGY2, Winooski, VT, USA).

Photothermal Performances: An 808 nm NIR laser (LWIRPD-5, Beijing Laserwave OptoElectronics Tech. Co., Ltd., Beijing, China) was used to evaluate the photothermal properties of 5A-Gel. Gel0, 5A-Gel20, 5A-Gel50 and 5A-Gel70 were initially subjected to the irradiation of the 808 nm laser at the same power density (3 W) for a duration of 3 min. 5A-Gel50 was exposed to the irradiation of the 808 nm laser at varying power densities for 3 min. Throughout the irradiation process, thermal images and temperature of samples were captured by an infrared thermal camera (FLIR).

Hemocompatibility Evaluation: Fresh mouse whole blood was diluted with normal saline to an 8% (*v*/*v*) final concentration. The 5A-Gel hydrogel served as the experimental group, while PBS and 1% Triton X-100 were employed as negative and positive controls, respectively. Briefly, 1 mL of the diluted blood was added to centrifuge tubes containing either 5A-Gel, PBS, or Triton X-100. The tubes were then kept in a water bath at 37 °C for 30 min. Following incubation, the tubes were centrifuged at 1500 rpm for 5 min. Finally, the supernatants were collected after centrifugation, and the absorbance was read at 570 nm using a microplate reader.

In vivo Frostbite Healing Experiments: The frostbite model in ICR mice was established based on a previously reported method [9]. Male ICR mice aged 6–8 weeks and weighing 25–30 g were anesthetized. The dorsal hair was shaved using an electric clipper followed by depilatory cream. A round iron rod (10 mm in diameter) was pre-cooled on dry ice for 10 min and then placed in close contact with the mouse back for 1 min to induce a frostbite wound. Then, the frostbite wound was covered with either nothing, 5A-Gel50 alone, or 5A-Gel50 with photothermal therapy (*n* = 5). All histological staining, imaging and morphological scoring were completed by two independent researchers under blinded grouping conditions. For the photothermal treatment groups (5A-Gel50+), the wound site was subsequently exposed to irradiation from an 808 nm NIR laser for 30 min per day over three consecutive days. The thermal images of the wound site were captured using an infrared thermal camera. The macroscopic appearance of the underlying skin was photographed.

On day 3 post-surgery, the mice were sacrificed, and the skin tissues including the entire wound and margin of surrounding healthy skin were harvested. These samples were subsequently fixed in 4% paraformaldehyde, embedded in paraffin, and sliced into 5 μm thick sections. The resulting sections underwent H&E staining, Masson’s trichrome staining, and immunofluorescence labeling for IL-6 and CD31. All histological evaluations were performed in a blinded manner by two independent observers.

Statistical analysis: All data are expressed as mean ± standard deviation (SD). Intergroup differences were evaluated using the t-test for two groups, and one-way analysis of variance (ANOVA) for multiple groups. Statistical significance was defined as *p* < 0.05 (with * *p* < 0.05, ** *p* < 0.01, and *** *p* < 0.001).

## Data Availability

The original contributions presented in the study are included in the article; further inquiries can be directed to the corresponding author.

## Abbreviations

The following abbreviations are used in this manuscript:

TPPs	Tea polyphenols
ROS	Reactive oxygen species
PBS	Phosphate-buffered saline
IL-6	Interleukin-6
NIR	Near-infrared ray
